# Supplementary material for: Different types of theta rhythmicity are induced by social and fearful stimuli in a network associated with social memory
Source: eLife. 2015 Feb 16;4:e03614. doi: 10.7554/eLife.03614 (PMC4353977; doi:10.7554/eLife.03614)
Supplement: Figure 3—source data 1. — One-way ANOVA (repeated measures) test was used to determine whether there is a significant difference between the mean ΔTP of all five encounters during either social (1a) or object (1b) recognition. The assumption of normality was assessed by Lilliefors and Shapiro–Wilk tests. Sphericity was assessed by Mauchly's test. DOI: http://dx.doi.org/10.7554/eLife.03614.007 [file elife03614s001.docx]

**Figure 3 – source data 1**

| **Figure 3 – source data 1a** – Social Recognition; ANOVA - (Fig. 3b) | | | | | | |
| --- | --- | --- | --- | --- | --- | --- |
|  | **Sig. of Mauchly's Test** | **n** | ***p*** | **F** | **df** | **Correction** |
|  |  |  |  |  |  |  |
| **AOB** | 0.142 | 8 | **<0.0001** | 7.088 | 4 | - |
| **MOB** | 0.179 | 8 | **<0.0001** | 9.648 | 4 | - |
| **MeAV** | 0.159 | 7 | **<0.005** | 5.734 | 4 | - |
| **LS** | 0.115 | 6 | **<0.005** | 5.369 | 4 | - |
| **Pir** | 0.225 | 6 | **<0.005** | 5.231 | 4 | - |
| **IT** | 0.772 | 8 | **<0.0001** | 8.336 | 4 | - |

| **Figure 3 – source data 1b** – Object Recognition; ANOVA (Fig. 3b) | | | | | | |
| --- | --- | --- | --- | --- | --- | --- |
|  | **Sig. of Mauchly's Test** | **n** | ***p*** | **F** | **df** | **Correction** |
|  |  |  |  |  |  |  |
| **AOB** | 0.246 | 6 | >0.1 | 2.208 | 4 | - |
| **MOB** | 0.309 | 6 | >0.05 | 2.34 | 4 | - |
| **MeAV** | 0.687 | 6 | >0.1 | 1.198 | 4 | - |
| **LS** | 0.314 | 6 | >0.1 | 0.984 | 4 | - |
| **Pir** | 0.041 | 6 | >0.1 | 1.393 | 4 | * |
| **IT** | 0.447 | 6 | **<0.0001** | 11.819 | 4 | - |

* Greenhouse-Geisser or Huynh-Feldt corrections were applied if applicable (Mauchly's test *p*<0.05).

**Figure 3 – source data 1: Theta power (TP) modulation between encounters.**

One-way ANOVA (repeated measures) test was used to determine whether there is a significant difference between the mean ΔTP of all 5 encounters during either social (**1a**) or object (**1b**) recognition. The assumption of normality was assessed by Lilliefors and Shapiro-Wilk tests. Sphericity was assessed by Mauchly's test.
