# Supplementary material for: Different types of theta rhythmicity are induced by social and fearful stimuli in a network associated with social memory
Source: eLife. 2015 Feb 16;4:e03614. doi: 10.7554/eLife.03614 (PMC4353977; doi:10.7554/eLife.03614)
Supplement: Figure 3—source data 2. — Paired t-tests were used for the social (2a) and object (2b) recognition paradigms, to examine if the differences between Enc.1 and Enc. 4 (habituation), as well as between Enc. 4 and Enc. 5 (dishabituation) are statistically significant. Tests were one-sided and corrected for multiple comparisons using Bonferroni's correction. DOI: http://dx.doi.org/10.7554/eLife.03614.008 [file elife03614s002.docx]

**Figure 3 – source data** **2**

| **Figure 3 – source data 2a** – Social Recognition; one-sided corrected paired t-tests - (Fig. 3b) | | | | | | | | |
| --- | --- | --- | --- | --- | --- | --- | --- | --- |
| **n** | **Paradigm** | **Region** | **t** | ***p*_corr_** | **Paradigm** | **Region** | **t** | ***p*_corr_** |
| 8 | **Habituation:**  **Enc. 1 vs. Enc. 4** | AOB | 3.497 | **<0.01** | **Dishabituation:**  **Enc. 4 vs. Enc. 5** | AOB | -3.737 | **<0.01** |
| 8 |  | MOB | 4.538 | **<0.005** |  | MOB | -3.502 | **<0.01** |
| 7 |  | MeA | 3.184 | **<0.05** |  | MeA | -3.049 | **<0.05** |
| 6 |  | LS | 2.842 | **<0.05** |  | LS | -4.04 | **<0.01** |
| 6 |  | Pir | 2.804 | **<0.05** |  | Pir | -6.485 | **<0.001** |
| 8 |  | IT | 5.931 | **<0.001** |  | IT | -3.465 | **<0.01** |

| **Figure 3 – source data 2b** – Object Recognition; one-sided corrected paired t-test - (Fig. 3b) | | | | | | | | |
| --- | --- | --- | --- | --- | --- | --- | --- | --- |
| n/df | **Paradigm** | **Region** | **t** | ***p*_corr_** | **Paradigm** | **Region** | **t** | ***p*_corr_** |
| 5 | **Habituation:**  **Enc. 1 vs. Enc. 4** | AOB | 2.228 | >0.05 | **Dishabituation:**  **Enc. 4 vs. Enc. 5** | AOB | -1.106 | >0.05 |
| 5 |  | MOB | 2.231 | >0.05 |  | MOB | -1.543 | >0.05 |
| 5 |  | MeA | 1.293 | >0.05 |  | MeA | 0.068 | >0.05 |
| 5 |  | LS | 0.432 | >0.05 |  | LS | 0.697 | >0.05 |
| 5 |  | Pir | 1.238 | >0.05 |  | Pir | -0.664 | >0.05 |
| 5 |  | IT | 4.591 | **<0.01** |  | IT | -3.941 | **<0.02** |

**Figure 3 – source data 2: Statistical assessment of habituation and dishabituation**

Paired t-tests were used for the social (**2a**) and object (**2b**) recognition paradigms, to examine if the differences between Enc.1 and Enc. 4 (habituation), as well as between Enc. 4 and Enc. 5 (dishabituation) are statistically significant. Tests were one-sided and corrected for multiple comparisons using Bonferroni's correction.
