# Supplementary material for: Different types of theta rhythmicity are induced by social and fearful stimuli in a network associated with social memory
Source: eLife. 2015 Feb 16;4:e03614. doi: 10.7554/eLife.03614 (PMC4353977; doi:10.7554/eLife.03614)
Supplement: Figure 4—source data 1. — Paired t-tests were used to compare between the mean ΔTP across Enc. vs the mean ΔTP across Post periods. The assumption of normality was assessed by Lilliefors and Shapiro–Wilk tests. DOI: http://dx.doi.org/10.7554/eLife.03614.010 [file elife03614s003.docx]

**Figure 4 – source data 1**

| **Figure 4 – source data 1** - one-sided paired t-test - (Fig. 4c) | | | | |
| --- | --- | --- | --- | --- |
| **Paradigm** | **Region** | **n** | **t** | ***p*** |
| **Social Recognition** | **AOB** | 5 | -1.7361 | >0.05 |
|  | **MOB** | 5 | -1.2243 | >0.05 |
|  | **MeAV** | 5 | -0.7389 | >0.05 |
|  | **LS** | 4 | -1.3153 | >0.05 |
|  | **Pir** | 5 | 0.1271 | >0.05 |
| **Object Recognition** | **AOB** | 4 | -2.9281 | **<0.05** |
|  | **MOB** | 4 | -4.5422 | **<0.05** |
|  | **MeAV** | 4 | -2.6806 | **<0.05** |
|  | **LS** | 4 | -3.543 | **<0.05** |
|  | **Pir** | 4 | -2.9717 | **<0.05** |

**Figure 4 – source data 1: Comparison of ΔTP between Enc. and Post periods**

Paired t-tests were used to compare between the mean ΔTP across Enc. vs. the mean ΔTP across Post periods**.** The assumption of normality was assessed by Lilliefors and Shapiro-Wilk tests.
