# Supplementary material for: Different types of theta rhythmicity are induced by social and fearful stimuli in a network associated with social memory
Source: eLife. 2015 Feb 16;4:e03614. doi: 10.7554/eLife.03614 (PMC4353977; doi:10.7554/eLife.03614)
Supplement: Figure 6—source data 1. — The change from Base to Enc. 1 (upper) and from Base to Post 1 (lower), in theta coherence during social recognition between the MeA and all other areas (1a) and between the MOB and all areas (1b), as well as during object recognition between the MeA and all other areas (1c), and between the MOB and all areas (1d), was statistically validated using paired t-tests, corrected for multiple comparisons (Bonferroni correction). The assumption of normality was assessed by Lilliefors and Shapiro–Wilk tests. DOI: http://dx.doi.org/10.7554/eLife.03614.020 [file elife03614s004.docx]

**Figure 6 – source data 1**

| **Social recognition - MeA vs. All** | | | | |
| --- | --- | --- | --- | --- |
| **Figure 6 – source data 1a -** one-sided corrected paired t-test (Fig.6d) | | | | |
| **MeA** | **vs.** | **n** | **t** | **p** |
| **Base vs. Enc. 1** | **AOB** | 11 | -1.0045 | >0.05 |
|  | **MOB** | 11 | 0.924 | >0.05 |
|  | **LS** | 10 | -3.0458 | **<0.05** |
|  | **Pir** | 11 | -3.8628 | **<0.005** |
| **Base vs. Post 1** | **AOB** | 11 | -1.4723 | >0.05 |
|  | **MOB** | 11 | -0.3362 | >0.05 |
|  | **LS** | 10 | -2.3561 | **<0.05** |
|  | **Pir** | 11 | -2.6767 | **<0.03** |
| **Social recognition - MOB vs. All** | | | | |
| **Figure 6 – source data 1b -** one-sided corrected paired t-test (Fig.6e) | | | | |
| **MOB** | **vs.** | **n/df** | **t** | **p** |
| **Base vs. Enc. 1** | **AOB** | 11 | -3.9002 | **<0.005** |
|  | **MeA** | 11 | 0.924 | >0.05 |
|  | **LS** | 10 | 0.4468 | >0.05 |
|  | **Pir** | 11 | 0.1002 | >0.05 |
| **Base vs. Post 1** | **AOB** | 11 | -2.3061 | **<0.05** |
|  | **MeA** | 11 | -0.3362 | >0.05 |
|  | **LS** | 10 | -0.1999 | >0.05 |
|  | **Pir** | 11 | 0.3943 | >0.05 |
| **Object recognition - MeA vs. All** | | | | |
| **Figure 6 – source data 1c -** one-sided corrected paired t-test (Fig.6f) | | | | |
| **MeA** | **vs.** | **n** | **t** | **p** |
| **Base vs. Enc. 1** | **AOB** | 5 | -0.3898 | >0.05 |
|  | **MOB** | 5 | -0.295 | >0.05 |
|  | **LS** | 5 | -1.4958 | >0.05 |
|  | **Pir** | 5 | -1.8165 | >0.05 |
| **Base vs. Post 1** | **AOB** | 5 | -0.7966 | >0.05 |
|  | **MOB** | 5 | -0.7301 | >0.05 |
|  | **LS** | 5 | -1.1095 | >0.05 |
|  | **Pir** | 5 | -1.1095 | >0.05 |
| **Object recognition - MOB vs. All** | | | | |
| **Figure 6 – source data 1d -** one-sided corrected paired t-test (Fig.6g) | | | | |
| **MOB** | **vs.** | **n** | **t** | **p** |
| **Base vs. Enc. 1** | **AOB** | 5 | -4.8906 | **<0.005** |
|  | **LS** | 5 | 0.4877 | >0.05 |
|  | **Pir** | 5 | 0.4902 | >0.05 |
|  | **MeA** | 5 | -0.295 | >0.05 |
| **Base vs. Post 1** | **AOB** | 5 | -1.5652 | >0.05 |
|  | **LS** | 5 | 1.4481 | >0.05 |
|  | **Pir** | 5 | 0.4052 | <0.05 |
|  | **MeA** | 5 | -0.7301 | >0.05 |

**Figure 6 – source data 1: Assessment of change in theta Coherence from Base to either Enc. 1 or Post 1**

The change from Base to Enc. 1 (upper) and from Base to Post 1 (lower), in theta coherence during social recognition between the MeA and all other areas (**1a**) and between the MOB and all areas (**1b**), as well as during object recognition between the MeA and all other areas (**1c**), and between the MOB and all areas (**1d**), was statistically validated using paired t-tests, corrected for multiple comparisons (Bonferroni correction). The assumption of normality was assessed by Lilliefors and Shapiro-Wilk tests**.**
