# Supplementary material for: Different types of theta rhythmicity are induced by social and fearful stimuli in a network associated with social memory
Source: eLife. 2015 Feb 16;4:e03614. doi: 10.7554/eLife.03614 (PMC4353977; doi:10.7554/eLife.03614)
Supplement: Figure 7—source data 1. — Comparison of the change in theta power between social recognition (SR) and fear conditioning (FC) at high and low theta ranges, statistically validated using two-way repeated measures ANOVA (p—experiment X theta range interaction). The assumption of normality was assessed by Lilliefors and Shapiro–Wilk tests. DOI: http://dx.doi.org/10.7554/eLife.03614.025 [file elife03614s005.docx]

**Figure 7 – source data 1**

| **Figure 7 – source data 1 -** two-way repeated measures ANOVA- Fig.7d | | | | |
| --- | --- | --- | --- | --- |
|  | **n** | **df** | **F** | ***p*** |
| **AOB** | 5 | 1,4 | 65.160 | <0. 01 |
| **MOB** | 6 | 1,5 | 47.95 | <0. 01 |
| **Nacc** | 5 | 1,4 | 34.63 | <0. 01 |
| **Pir** | 6 | 1,5 | 39.29 | <0. 01 |
| **LS** | 6 | 1,5 | 24.39 | <0. 01 |
| **MeA** | 6 | 1,5 | 26.63 | <0. 01 |

**Figure 7 – source data 1: Comparison of change in theta power in low and high theta bands between social and fearful stimuli.**

Comparison of the change in theta power between social recognition (SR) and fear conditioning (FC) at high and low theta ranges, statistically validated using two-way repeated measures ANOVA (*p* - experiment X theta range interaction). The assumption of normality was assessed by Lilliefors and Shapiro-Wilk tests**.**
