# Supplementary material for: Different types of theta rhythmicity are induced by social and fearful stimuli in a network associated with social memory
Source: eLife. 2015 Feb 16;4:e03614. doi: 10.7554/eLife.03614 (PMC4353977; doi:10.7554/eLife.03614)
Supplement: Figure 8—source data 1. — Comparison of the change in coherence between social recognition (SR) and fear conditioning (FC) at high and low theta ranges (right), statistically validated using two-way repeated measures ANOVA (p—experiment X theta range interaction). The assumption of normality was assessed by Lilliefors and Shapiro–Wilk tests. DOI: http://dx.doi.org/10.7554/eLife.03614.028 [file elife03614s006.docx]

**Figure 8 – source data 1**

| **Figure 8– source data 1 -** two-way repeated measures ANOVA - Fig.8c | | | | | |
| --- | --- | --- | --- | --- | --- |
| **AOB vs.** |  | **n** | **df** | **F** | ***p*** |
|  | **MOB** | 5 | 1,4 | 80.92 | <0. 01 |
|  | **Nacc** | 4 | 1,3 | 9.82 | >0.05 |
|  | **Pir** | 5 | 1,4 | 31.54 | <0. 01 |
|  | **LS** | 5 | 1,4 | 21.90 | <0. 01 |
|  | **MeA** | 5 | 1,4 | 19.89 | <0.05 |
| **MOB vs.** | **Nacc** | 5 | 1,4 | 17.24 | <0.05 |
|  | **Pir** | 6 | 1,5 | 24.61 | <0. 01 |
|  | **LS** | 6 | 1,5 | 26.19 | <0. 01 |
|  | **MeA** | 6 | 1,5 | 7.19 | <0.05 |
| **Nacc vs.** | **Pir** | 5 | 1,4 | 11.72 | <0.05 |
|  | **LS** | 5 | 1,4 | 6.54 | >0.05 |
|  | **MeA** | 5 | 1,4 | 26.69 | <0. 01 |
| **Pir vs.** | **LS** | 6 | 1,5 | 24.39 | <0. 01 |
|  | **MeA** | 6 | 1,5 | 15.58 | <0. 01 |
| **LS vs.** | **MeA** | 6 | 1,5 | 18.52 | <0. 01 |

**Figure 8 – source data 1: Comparison of change in coherence in low and high theta bands between social and fearful stimuli.**

Comparison of the change in coherence between social recognition (SR) and fear conditioning (FC) at high and low theta ranges (right), statistically validated using two-way repeated measures ANOVA (*p* - experiment X theta range interaction). The assumption of normality was assessed by Lilliefors and Shapiro-Wilk tests**.**
